# Upper Airway Changes after Orthodontic Extraction Treatment in Adults: A Preliminary Study using Cone Beam Computed Tomography

**DOI:** 10.1371/journal.pone.0143233

**Published:** 2015-11-20

**Authors:** Jingjing Zhang, Gui Chen, Weiran Li, Tianmin Xu, Xuemei Gao

**Affiliations:** Department of Orthodontics, Peking University School and Hospital of Stomatology, Beijing, PR China; Medical University of South Carolina, UNITED STATES

## Abstract

**Objective:**

Whether the orthodontic treatment with premolar extraction and maximum anchorage in adults will lead to a narrowed upper airway remains under debated. The study aims to investigate the airway changes after orthodontic extraction treatment in adult patients with Class II and hyperdivergent skeletal malocclusion.

**Materials and Methods:**

This retrospective study enrolled 18 adults with Class II and hyperdivergent skeletal malocclusion (5 males and 13 females, 24.1 ± 3.8 years of age, BMI 20.33 ± 1.77 kg/m^2^). And 18 untreated controls were matched 1:1 with the treated patients for age, sex, BMI, and skeletal pattern. CBCT images before and after treatment were obtained. DOLPHIN 11.7 software was used to reconstruct and measure the airway size, hyoid position, and craniofacial structures. Changes in the airway and craniofacial parameters from pre to post treatment were assessed by Wilcoxon signed rank test. Mann-Whitney U test was used in comparisons of the airway parameters between the treated patients and the untreated controls. Significant level was set at 0.05.

**Results:**

The upper and lower incisors retracted 7.87 mm and 6.10 mm based on the measurement of U1-VRL and L1-VRL (P < 0.01), while the positions of the upper and lower molars (U6-VRL, and L6-VRL) remained stable. Volume, height, and cross-sectional area of the airway were not significantly changed after treatment, while the sagittal dimensions of SPP-SPPW, U-MPW, PAS, and V-LPW were significantly decreased (P < 0.05), and the morphology of the cross sections passing through SPP-SPPW, U-MPW, PAS, and V-LPW became anteroposteriorly compressed (P <0.001). No significant differences in the airway volume, height, and cross-sectional area were found between the treated patients and untreated controls.

**Conclusions:**

The airway changes after orthodontic treatment with premolar extraction and maximum anchorage in adults are mainly morphological changes with anteroposterior dimension compressed in airway cross sections, rather than a decrease in size.

## Introduction

Since Angle reported a narrowed upper airway in children with Class II dentofacial deformity in 1907 [1], many studies have demonstrated that certain craniofacial patterns are related with a smaller size of the upper airway, including deficient mandible, and steep mandibular plane [2–4]. Individuals with Class II deformity and steep mandibular plane possess a smaller airway, and are higher risks for obstructive sleep apnea (OSA), especially Asians [5, 6].

Individuals with Class II and hyperdivergent skeletal pattern usually present with a convex profile, which is often their chief complaint in orthodontic clinics. Orthognathic surgery can completely address the problems of abnormal profile and narrowed upper airway [7–9]. But most patients regard it as too aggressive, and thus reject the treatment. Orthodontic camouflage treatment can improve the profile in those with mild to moderate skeletal discrepancy, usually by means of teeth extraction and maximum anchorage [10–12]. However, whether this approach will affect the size of the upper airway remains a matter of debate.

Wang et al and Chen et al reported a decreased airway size after orthodontic extraction treatment with maximum anchorage in adult patients without skeletal discrepancy, and they also found that the decreased airway size was correlated with the retraction amount of the lower incisors [13, 14]. But Maaitah et al found unchanged airway size in his study [15]. We found few studies were done in the patients with Class II skeletal malocclusion except that Kikuchi published a case report of decreased airway dimension after orthodontic extraction treatment in a girl with Class II malocclusion [16]. The possible mechanism has been concluded as the decreased oral cavity size, and the influenced position of the tongue and hyoid bone. Since the airway changes and the possible mechanism remain under debated, it is worth concerned what effect could be exerted on the upper airway by orthodontic extraction treatment, especially in patients with Class II malocclusion.

Most previous studies have used lateral cephalograms, which provide two-dimensional (2D) images. Given the advantages of a relatively low dose of radiation and comprehensive three-dimensional (3D) images, CBCT has been used for airway analysis in recent years and measurements of the upper airway and craniofacial structures have been demonstrated to be precise and reliable [17–19].

The present study was carried out retrospectively in adult patients with Class II and hyperdivergent skeletal pattern using cone beam computed tomography (CBCT). The null hypothesis was that the size of the upper airway was not changed after the orthodontic camouflage treatment with extraction of four premolars and maximum anchorage. To explore the possible clinical importance of the changes in the airway size, an untreated control group of skeletal matched patients was compared to the treated patients.

## Materials and Methods

### Patient Selection

Patients were selected from the existing database of the Department of Orthodontics, Peking University School and Hospital of Stomatology. The time of first referral was between January 2009 and September 2009. The study protocol was approved by Ethics Committee of Peking University School and Hospital of Stomatology (PKUSSIRB-201520022). All data were re-numbered and analyzed anonymously.

Inclusion criteria were: 1) age ≥ 18 years; 2) sagittal Class II (ANB > 4.7°) and vertical hyperdivergent (MP/SN > 37.7°) skeletal pattern; 3) convex profile evaluated by E line; 4) no missing teeth except for the third molars; 5) orthodontic camouflage treatment with extraction of four premolars and maximum anchorage using mini-screws; 6) available CBCT data both before and after treatment. Exclusion criteria were: 1) body mass index (BMI) ≥ 25 kg/m^2^; 2) specific approaches involved in the treatment, including rapid maxillary expansion, protraction facemask therapy, extra oral force to push molars distally, functional appliances, and orthognathic surgery [20–25]; 3) history of cleft lip or palate; 4) hyperplasia of tonsils or adenoids, or history of tonsillectomy or adenoidectomy; 5) snoring or other sleep disorders.

Eighteen patients (5 males and 13 females), 24.1 ± 3.8 years of age (range of 18–33 years) with a BMI = 20.33 ± 1.77 kg/m^2^, were included in the analysis. All of them were treated by the same orthodontist. Thirteen patients had a Class II molar relationships from 1/2 unit Class II to full unit Class II, and five patients had a slight Class II molar relationships. The canine relationships were all Class II. Crowding in the upper and lower arches were mild crowding (less than 4 mm), and mild to moderate crowding (less than 7 mm), respectively. Twelve patients experienced extraction of four first premolars, while the other six experienced extraction of two upper first premolars and two lower second premolars. The treatment period varied from 18 to 36 months, with mean of 30.0 months.

Eighteen untreated patients were used as controls and matched 1:1 with the treated patients at the time point when the treatment ended. Patients were matched for age, sex, BMI (differences less than 1 kg/m^2^), sagittal skeletal pattern (ANB, differences less than 1°), and vertical skeletal pattern (MP/SN, differences less than 1°). CBCT data before any treatment were used in the analysis. Six of them did take another CBCT when the treatment ended, ten only took panoramic films and lateral cephalograms, and the other two were still wearing braces when the study was carried out.

### Acquisition of CBCT Images

All CBCT images before and after treatment were obtained with the same CBCT scanner (DCT PRO Dentofacial CBCT System, VATECH, Korea) according to a standard protocol (90 kV, 7 mA, 20 cm×19 cm FOV, 0.40 mm voxel resolution, and 15 s scan time), and performed by the same operator. Patients were instructed to sit upright with a natural head position, maximum intercuspation of the teeth, normal respiration, and no swallowing during the scanning process. The Frankfort planes were adjusted parallel to the ground. And they were instructed to maintain the rest position of the tongue, that is, in contact with the anterior hard palate and no touch the anterior teeth. The datasets were exported in DICOM (Digital Imaging and Communications in Medicine) format, and then transferred into a Dolphin 11.7 software package (Dolphin Imaging & Management Solutions, Chatsworth, CA). The software automatically reconstructed and identified the craniofacial structures, upper airway, hyoid bone, and vertebrae based on Hounsfield units (HU). The HU ranged from 188 to 922 for the hard tissue, and from -3204 to -1000 for the upper airway.

### Measurements of the Upper Airway and Craniofacial Structures

All the measurements were done by the same researcher within 1 month using the Dolphin software. The time points were labeled T0 (before treatment) and T1 (immediately after treatment).

**Fig 1:**
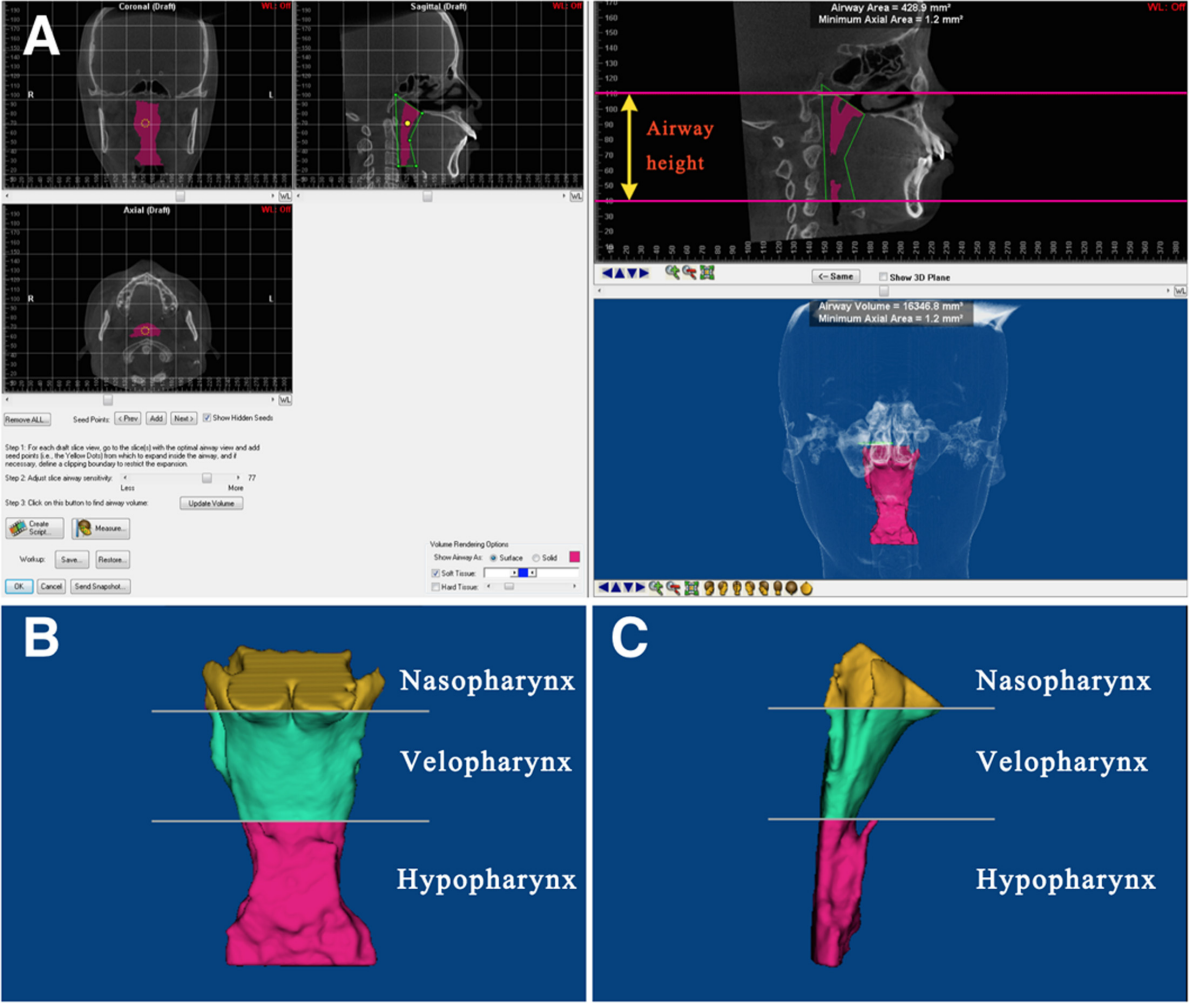
Airway measurements of the volume (V), height (H), and minimum cross sectional area (Min) using Dolphin 11.7 software package. (A). Pink area defines the airway portion of interest, and the green plane locates the minimum cross sectional area. V, H, and Min were automatically calculated; (B). Front view of the evaluated upper airway. The airway was divided into nasopharynx, velopharynx, and hypopharynx by two horizontal planes passing the posterior nasal spine and the tip of the soft palate; (C). Lateral view of the evaluated upper airway.

The upper airway in the CBCT images was analyzed from the top of the airway to the horizontal level of the C3 point (the most anterior and inferior point of the third cervical vertebra), and it was divided into three parts, nasopharynx, velopharynx, and hypopharynx, according to the corresponding cross-sectional slices. The nasopharynx (Naso-) was defined as the region from the top of the airway to the plane passing the posterior nasal spine; the velopharynx (Velo-) as the region from the posterior nasal spine to the tip of the soft palate; and the hypopharynx (Hy-) as the lowest region to the level of the C3 point. Each region was reconstructed and measured using the Dolphin 11.7 software. The parameters were airway volume (V), airway height (H), minimum cross-sectional area (Min), and mean cross-sectional area (Mean). The software automatically calculated V, H, and Min of each region (Fig 1). Mean was computed as the V/H ratio.

**Fig 2:**
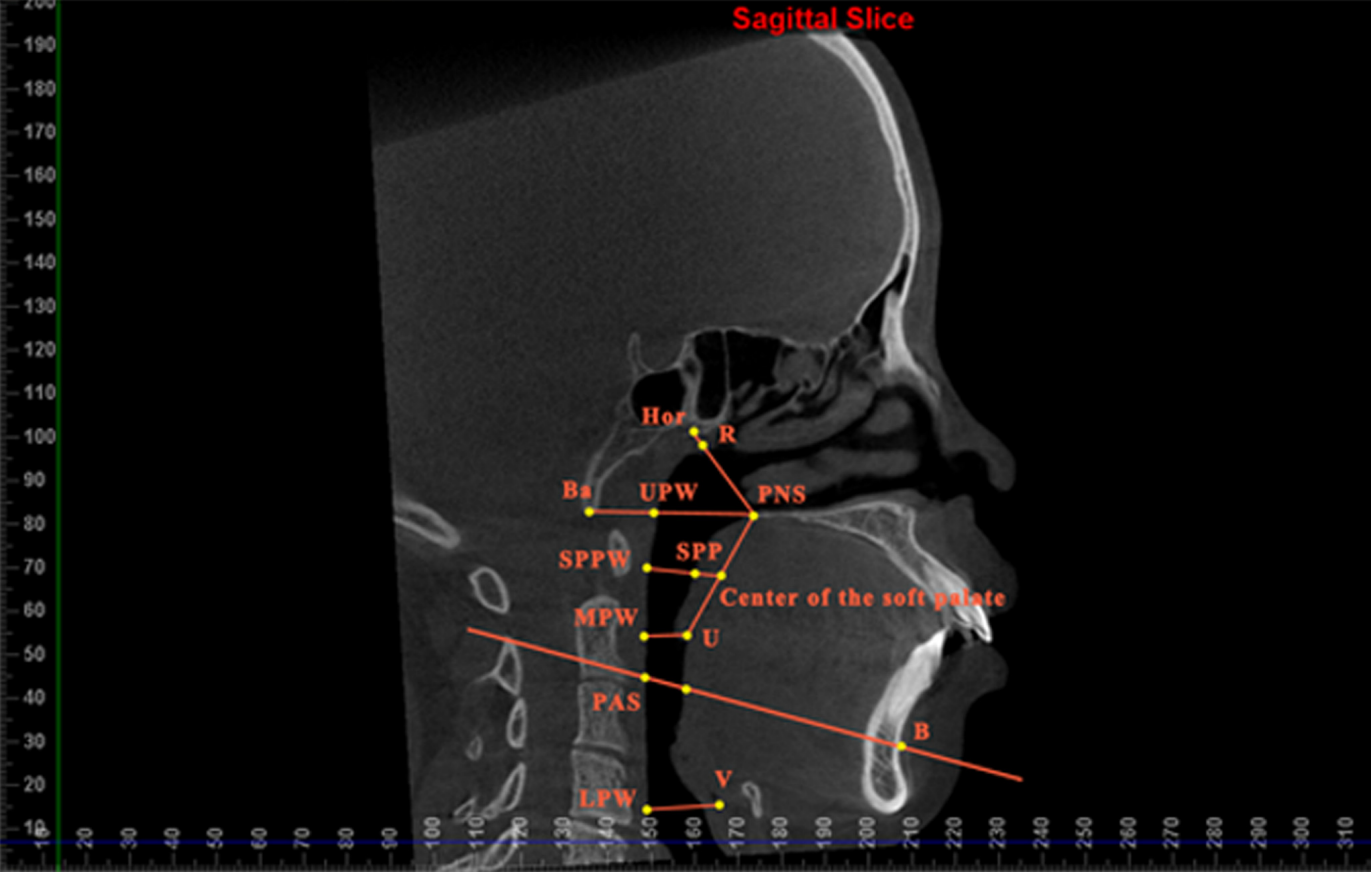
Measurements of the sagittal airway dimension in the mid-sagittal plane of CBCT.

**Fig 3:**
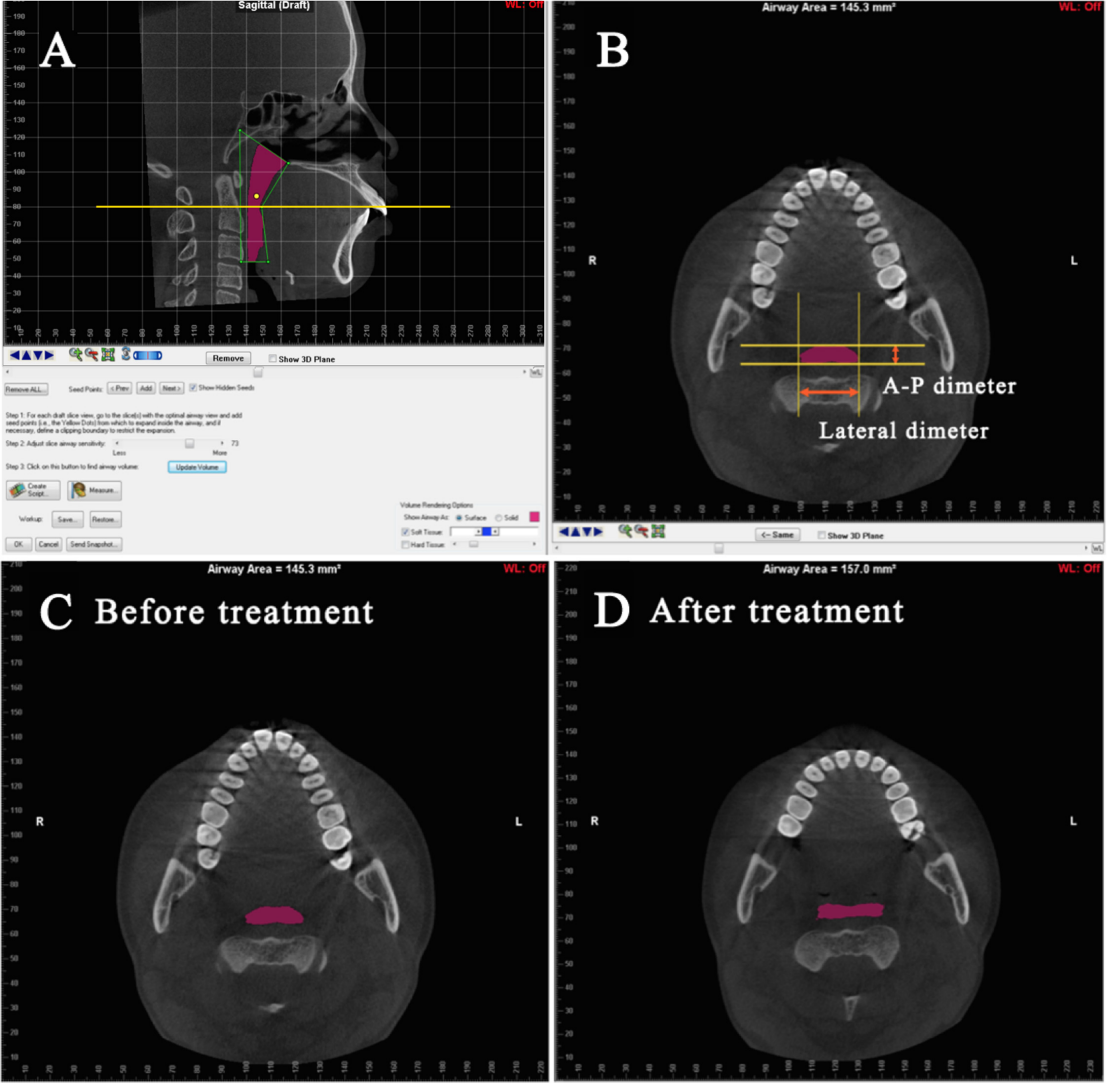
Measurements of the area and morphology of the cross-sectional planes passing the sagittal linear measurements. (A) Pink area defines the upper airway, and the yellow line indicates the plane passing the sagittal airway parameter of U-MPW. (B) Coronal view of the cross section passing the U-MPW. The A-P dimeter, lateral dimeter, and area are measured. (C) and (D) showed the typical changes of the morphology from pre to post treatment in the same cross section passing U-MPW.

For better comparisons with previous studies using lateral cephalograms, the sagittal dimensions of the upper airway were also measured. Measurements were done in the mid-sagittal plane in the CBCT images, and six parameters were selected (Table 1 and Fig 2). Cross-sectional planes passing the sagittal parameters were reconstructed and reoriented using the Dolphin software, and several measurements, the cross-sectional area, A-P diameter, lateral diameter, and ratio of A-P to lateral diameter (Ratio), were measured in these planes (Fig 3A and 3B). The parameter Ratio provides an index of the circularity in the upper airway, with a ratio of 1.0 representing a circle, a ratio < 1.0 representing an ellipse with the long axis oriented laterally, and a ratio > 1.0 representing an ellipse with the long axis oriented in the A-P dimension.

**Table 1:** Measurements of the sagittal airway dimension, craniofacial structures, and hyoid position.

**Parameters of the airway sagittal dimension, measured in the mid-sagittal plane of CBCT, mm**	
**PNS-R**	Distance between PNS and R (point locates at the intersection between posterior pharyngeal wall and PNS-Hor line)
**PNS-UPW**	Distance between PNS and UPW (point locates at the intersection between posterior pharyngeal wall and PNS-Ba line)
**SPP-SPPW**	Distance between SPP (point of intersection of line from soft palate center perpendicular to posterior pharyngeal wall and posterior margin of soft palate) and SPPW (point of intersection of line from soft palate center perpendicular to posterior pharyngeal wall and posterior pharyngeal wall)
**U-MPW**	Distance between U (tip of soft palate) and MPW (foot point at the posterior pharyngeal wall of perpendicular line from point U)
**PAS**	Width of the airway space along the Go-B line
**V-LPW**	Distance between V (base of the epiglottis) and LPW (foot point at the posterior pharyngeal wall of perpendicular line from point V)
**Skeletal parameters, measured in the lateral cephalograms generated by CBCT**	
**SNA, degrees**	Angle between subspinale and sella at nasion, representing the position of the maxilla in relation to the cranium
**SNB, degrees**	Angle between supraemental and sella at nasion, representing the position of the mandible in relation to the cranium
**ANB, degrees**	Angle between subspinale and supraemental at nasion, representing the relationship of maxilla and mandible in relation to the cranium
**MP/SN, degrees**	Angle between the mandibular plane and SN plane, representing the mandibular inclination
**A-VRL, mm**	Horizontal distance from subspinale to a line drawn perpendicularly to Frankfort plane at S (VRL)
**B- VRL, mm**	Horizontal distance from supraemental to VRL
**Dental parameters, measured in the lateral cephalograms generated by CBCT**	
**U1/SN, degrees**	Angle between the long axis of the upper central incisor and SN plane
**U1- VRL, mm**	Horizontal distance from the tip of the upper central incisor to VRL
**L1/MP, degrees**	Angle between the long axis of the mesial lower incisor and the mandibular plane
**L1- VRL, mm**	Horizontal distance from the tip of the mesial lower incisor to VRL
**U6/SN, degrees**	Angle between the long axis of upper first molars and SN plane, representing the inclination of the upper molars
**U6/PP, degrees**	Angle between the long axis of upper first molars and the line extending from anterior nasion spine to posterior nasion spine, representing the inclination of the upper molars
**U6- VRL, mm**	Horizontal distance from the mesial buccal cusps of the upper first molars to VRL
**L6/MP, degrees**	Angle between the long axis of the lower first molars and the mandibular plane, representing the inclination of the lower molars
**L6- VRL, mm**	Horizontal distance from the mesial buccal cusps of the lower first molars to VRL
**Hyoid position, measured in the lateral cephalograms generated by CBCT, mm**	
**H-MP**	Perpendicular distance from H (the most superior and anterior point on the body of hyoid bone) to mandibular plane
**H-C3**	Distance between H and C3
**H-Rgn**	Distance between H and Rgn
**H-HRL**	Perpendicular distance from H to Frankfort plane (HRL)

**Fig 4:**
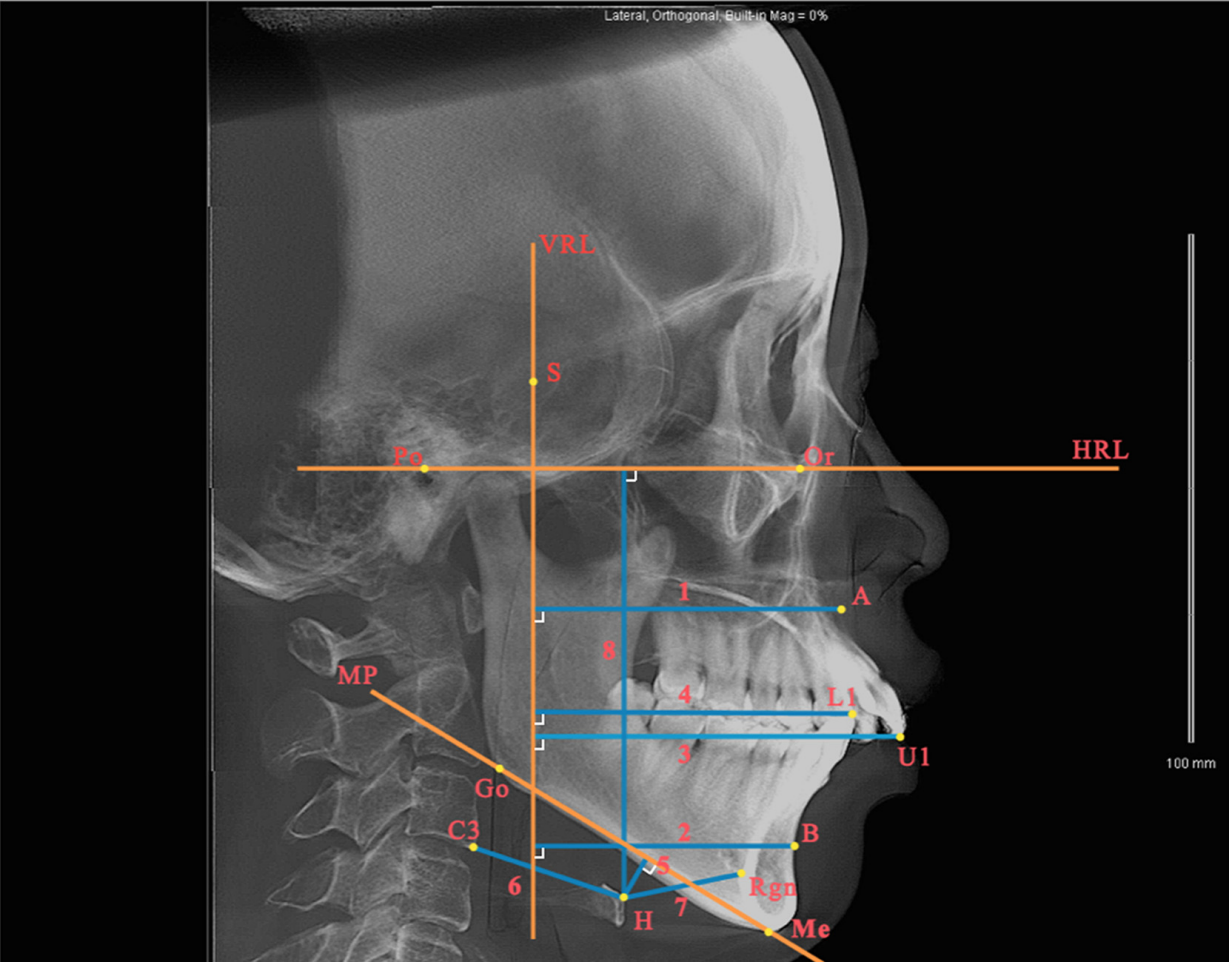
Measurements of the craniofacial structures and hyoid position in the lateral cephalograms generated by CBCT. (1) A-VRL; (2) B-VRL; (3) U1-VRL; (4) L1-VRL; (5) H-MP; (6) H-C3; (7) H-Rgn; and (8) H-HRL.

Measurements of the craniofacial structures and the position of the hyoid bone were done in the lateral cephalograms generated by CBCT (Table 1 and Fig 4). The Frankfort Horizontal Plane was used as the *x*-axis (HRL), and a perpendicular line (VRL) passing through the S point served as the *y*-axis.

### Error of the Method and Statistical Analysis

Nine participants were randomly selected for reliability testing. All measurements of the selected patients were rechecked 2 weeks later by the same researcher. The intra-class correlation coefficient was 0.958 (P < 0.001). The method error (ME) was calculated as: ME = (∑d^2^/2n) ^½^ (where d is deviation between the two measurements and n is the number of paired double measurements) [26]. ME varied from 0.01 to 0.23 mm for linear measurements, from 0.01 to 0.31° for angular measurements, from 4.8 to 14.2 mm^2^ for area measurements, and from 22 to 96 mm^3^ for volume measurements.

Statistical analysis was performed using the SPSS 16.0 (Statistical Product and Service Solutions, SPSS Inc., Chicago, IL). The power of test was 0.68. The Kolmogorov-Smirnov test was used to examine normality of the data, and the results showed that the distributions of all measurements were not normal, and thus non-parametric tests were used. Changes of the airway and craniofacial measurements from T0 to T1 were assessed by Wilcoxon signed rank test. Mann-Whitney U test was used in the comparison of the airway measurements between the treated patients at T1 and the control group at T0. The significant level was set at 0.05.

## Results

Though no significant changes of the skeletal measurements from T0 to T1 were found, changes in the dental measurements were significant (Table 2 and S1 File). The upper incisors retracted 7.87 mm based on the measurement of U1-VRL, and the lower incisors 6.10 mm based on L1-VRL (P < 0.01). The positions of the upper and lower molars (U6-VRL, and L6-VRL) were not significantly changed from T0 to T1, but they both inclined clockwise at T1 compared with T0 (P < 0.05). No significant changes from T0 to T1 were found in the position of the hyoid bone (Table 2 and S1 File).

**Table 2:** Changes in the craniofacial structures and position of the hyoid bone after orthodontic extraction treatment in adults with Class II and hyperdivergent pattern (n = 18).

	T0			T1			P Value^a^
	P25	P50	P75	P25	P50	P75	
**Skeletal Measurements**							
**SNA/°**	80.13	83.00	87.30	80.20	84.58	86.03	0.815
**SNB/°**	74.93	80.40	81.28	75.90	79.30	81.33	0.815
**ANB/°**	4.80	5.25	5.78	4.28	5.13	5.86	0.815
**MP/SN/°**	37.95	39.95	42.74	37.81	39.45	42.05	0.481
**A-VRL/mm**	59.83	61.10	66.00	59.11	60.83	65.55	0.663
**B- VRL /mm**	52.28	56.20	59.58	51.80	53.93	58.25	0.231
**Dental Measurements**							
**U1/SN/°**	105.83	108.93	113.41	87.90	91.85	98.88	0.000**
**U1- VRL /mm**	67.38	71.80	75.20	61.00	63.93	69.54	0.000**
**L1/MP/°**	91.75	97.00	100.18	87.90	91.45	96.58	0.002**
**L1- VRL /mm**	63.23	68.55	70.65	58.78	62.45	67.30	0.000**
**U6/SN/°**	71.08	75.65	80.53	67.55	73.40	77.15	0.002**
**U6/PP/°**	80.18	85.13	89.93	77.39	82.00	86.23	0.020*
**U6- VRL /mm**	40.19	42.30	46.08	40.25	42.03	45.53	0.983
**L6/MP/°**	93.45	98.20	99.88	96.90	100.90	103.23	0.026*
**L6- VRL /mm**	40.45	43.00	47.55	40.45	43.50	48.60	0.218
**Position of hyoid bone, mm**							
**H-MP**	6.10	12.45	13.98	4.86	10.25	13.53	0.728
**H-C3**	29.23	32.05	34.45	29.69	31.28	34.48	0.983
**H-Rgn**	29.20	31.00	34.33	27.80	29.30	35.40	0.948
**H-HRL**	76.48	82.95	85.90	75.50	82.00	85.28	0.931

* P<0.05

** P<0.01

^a^ Wilcoxon signed rank test

The volume, height, and cross-sectional area of the airway were not significantly changed from T0 to T1 (Table 3 and S2 File). However, the sagittal dimensions of SPP-SPPW, U-MPW, PAS, and V-LPW were significantly decreased at T1 compared to T0 (P < 0.05, Table 4 and S2 File).

**Table 3:** Changes in the volume, height, and cross sectional area of the upper airway after orthodontic extraction treatment in adults with Class II and hyperdivergent pattern (n = 18).

	T0			T1			P Value^a^
	P25	P50	P75	P25	P50	P75	
**Naso-V/mm** ^**3**^	4600	5580	6462	5017	5555	6843	0.145
**Naso-H/mm**	13.00	13.70	15.70	13.41	14.38	16.33	0.459
**Naso-Mean/mm** ^**2**^	328.0	381.0	431.0	350.5	396.5	424.6	0.811
**Velo-V/mm** ^**3**^	7110	9492	12653	8041	8779	12888	0.327
**Velo-H/mm**	23.46	25.60	27.75	23.38	26.20	27.24	0.711
**Velo-Min/mm** ^**2**^	136.2	218.2	339.1	161.3	229.9	323.3	0.112
**Velo-Mean/mm** ^**2**^	271.2	382.5	480.9	308.5	335.5	499.1	0.215
**Hy-V/mm** ^**3**^	5186	8577	12720	6721	8329	9797	0.306
**Hy-H/mm**	29.58	32.20	37.30	29.59	33.20	35.93	0.632
**Hy-Min/mm** ^**2**^	136.7	187.1	288.2	140.7	172.6	248.7	0.446
**Hy-Mean/mm** ^**2**^	187.3	260.9	335.0	199.5	237.2	303.6	0.500
**Total-V/mm** ^**3**^	16633	24008	29415	19065	22759	29546	0.102
**Total-H/mm**	70.08	75.83	78.13	69.54	75.83	78.80	0.616
**Total-Mean/mm** ^**2**^	239.2	340.1	377.8	263.9	308.9	389.2	0.199

* P<0.05

** P<0.01

^a^ Wilcoxon signed rank test

**Table 4:** Changes in the sagittal airway dimension and cross section morphology after orthodontic extraction treatment in adults with Class II and hyperdivergent pattern (n = 18).

Sagittal parameter		T0			T1			P Value^a^
		P25	P50	P75	P25	P50	P75	
**PNS-R**	**Sagittal dimension**	19.25	21.38	23.75	20.16	23.75	24.73	0.170
	**Area**	387.7	477.2	560.1	387.2	508.6	571.5	0.005^**^
	**A-P**	19.65	22.55	25.13	18.98	22.30	24.90	0.286
	**Lateral**	24.50	27.05	29.93	25.80	27.85	29.63	0.078
	**Ratio**	0.751	0.825	0.944	0.722	0.809	0.854	0.122
**PNS-UPW**	**Sagittal dimension**	21.88	24.70	26.35	22.53	26.35	26.98	0.338
	**Area**	524.3	628.7	702.0	526.8	668.1	752.2	0.327
	**A-P**	21.30	24.40	27.25	22.15	24.15	26.73	0.695
	**Lateral**	29.83	31.75	34.10	30.65	32.80	35.48	0.012^*^
	**Ratio**	0.699	0.768	0.857	0.614	0.721	0.795	0.102
**SPP-SPPW**	**Sagittal dimension**	10.70	12.90	14.75	10.50	14.75	12.70	0.006^**^
	**Area**	241.6	358.7	471.4	294.7	348.6	446.7	0.711
	**A-P**	11.10	14.35	16.40	9.93	11.15	13.80	0.000^**^
	**Lateral**	27.93	35.75	38.00	33.70	37.45	41.73	0.005^**^
	**Ratio**	0.377	0.412	0.459	0.268	0.298	0.378	0.000^**^
**U-MPW**	**Sagittal dimension**	8.50	11.15	14.01	7.73	14.01	12.29	0.012^*^
	**Area**	176.1	233.8	317.7	173.4	264.6	307.3	0.616
	**A-P**	10.05	11.25	13.65	6.95	9.75	11.53	0.002^**^
	**Lateral**	20.93	28.30	35.13	25.65	31.20	35.38	0.024^*^
	**Ratio**	0.367	0.421	0.516	0.258	0.340	0.392	0.000^**^
**PAS**	**Sagittal dimension**	9.56	10.88	13.18	7.90	13.18	10.23	0.003^**^
	**Area**	191.7	243.8	336.3	201.7	232.2	312.5	0.777
	**A-P**	9.90	11.40	13.70	7.38	8.65	12.10	0.002^**^
	**Lateral**	24.40	28.65	34.43	27.58	30.45	35.63	0.012^*^
	**Ratio**	0.387	0.419	0.483	0.253	0.314	0.347	0.000^**^
**V-LPW**	**Sagittal dimension**	13.10	15.00	16.38	12.38	16.38	16.00	0.012^*^
	**Area**	212.6	243.0	391.8	206.3	235.4	340.7	0.647
	**A-P**	9.83	13.10	15.43	7.23	10.60	12.73	0.000^**^
	**Lateral**	30.83	33.55	34.88	32.18	34.15	37.85	0.001^**^
	**Ratio**	0.311	0.376	0.470	0.223	0.300	0.372	0.000^**^

Area: area in the cross sections passing the corresponding sagittal parameter, mm^2^; A-P: A-P diameter, mm; Lateral: lateral diameter, mm; Ratio: ratio of A-P to lateral diameter

* P<0.05

** P<0.01

^a^ Wilcoxon signed rank test

Morphological changes in the cross sections of the upper airway are shown in Table 4 (S2 File). In the cross sections passing through SPP-SPPW, U-MPW, PAS, and V-LPW, the area remained stable from T0 to T1, while the A-P diameter decreased at T1 compared to T0 (P < 0.01), the lateral diameter increased (P < 0.05), and the Ratio decreased (P < 0.001).

The volume, height, and cross-sectional area of the upper airway in the treated patients at T1 were not significantly different from that of the matched untreated controls (Table 5 and S1 and S2 Files).

**Table 5:** Differences in the volume, height, and cross sectional area of the upper airway between the post-treatment adult patients and the matched untreated controls.

	Treated Group (n = 18)			Untreated Control Group (n = 18)			P Value^a^
	P25	P50	P75	P25	P50	P75	
**Naso-V/mm3**	5017	5555	6843	4433	5995	6643	0.975
**Naso-H/mm**	13.41	14.38	16.33	13.68	14.50	17.15	0.537
**Naso-Mean/mm2**	350.5	396.5	424.6	325.6	364.2	414.5	0.367
**Velo-V/mm3**	8041	8779	12888	8221	10484	12643	0.728
**Velo-H/mm**	23.38	26.20	27.24	24.05	27.10	29.15	0.223
**Velo-Min/mm2**	161.3	229.9	323.3	198.5	265.9	332.9	0.268
**Velo-Mean/mm2**	308.5	335.5	499.1	309.6	384.1	432.5	0.975
**Hy-V/mm3**	6721	8329	9797	6720	10053	13452	0.311
**Hy-H/mm**	29.59	33.20	35.93	31.30	35.35	38.58	0.217
**Hy-Min/mm2**	140.7	172.6	248.7	168.8	251.1	334.1	0.121
**Hy-Mean/mm2**	199.5	237.2	303.6	203.0	278.9	383.8	0.506
**Total-V/mm3**	19065	22759	29546	19976	26062	32842	0.327
**Total-H/mm**	69.54	75.83	78.80	71.95	79.65	83.90	0.107
**Total-Mean/mm2**	263.9	308.9	389.2	288.1	329.4	397.2	0.517

^a^ Mann-Whitney U test

## Discussion

Class II skeletal discrepancy and steep mandibular plane have been demonstrated to be associated with the decreased size of the upper airway, and an increased risk of OSA [2, 3, 27]. Orthodontic camouflage treatment for patients with mild to moderate skeletal discrepancy can solve the problems of mal-aligned teeth and unwanted profile [10, 11], but whether it will result in a decreased sized airway remains unknown. Previous studies reported that orthodontic extraction treatment could lead to a decreased airway size in adult patients [13, 14, 28], but most of them were done in those with no skeletal discrepancy. The present study was designed to add knowledge to the topic in the population with Class II and hyperdivergent skeletal pattern. Similar to the results of previous studies, the skeletal structures were not significantly changed after treatment, while the anterior teeth were markedly retracted [13–15, 28].

The effect of orthodontic extraction treatment on the upper airway seems to be an adaptive change in the airway morphology, rather than a decrease in the airway size (Fig 4B and 4C). After orthodontic treatment with premolar extraction and maximum anchorage, the airway volume, height, and cross-sectional area were not significantly changed. These findings are similar with those of Stefanovic [29], but different from those of Chen et al [13]. On the other hand, the sagittal airway dimensions decreased significantly in the middle and inferior parts, which are consistent findings with Wang et al, Sharma et al, and Germec-Cakan et al [14, 28, 30]. When looking at the cross sections passing the sagittal parameters, clues to the inconsistent results of decreased sagittal dimensions and unchanged volume, height, and cross-sectional area become apparent. With the morphological change to an ellipse compressed at the anteroposterior dimension, the area in the cross sections remained stable. CBCT can provide a comprehensive 3D view of the upper airway, while lateral cephalograms can only provide sagittal dimensions, which lead to completely different results. Such effects may remind us that self-regulation in the upper airway takes place during orthodontic treatment; that is, when the sagittal dimensions decreases, the lateral dimension increases to provide sufficient space for air passage. This theory, however, requires further investigation.

An untreated control group was used to eliminate the potential influence of normal growth, as development of the upper airway has been demonstrated to last until 20 years of age [31], and 5 patients in our study were under 20 when the treatment started. The control group was matched with the treated patients at T1 for age, sex, BMI, and skeletal pattern. Using this strategy, the effect of the orthodontic extraction treatment could be evaluated. It would be even better to have two sessions of CBCT in the untreated controls with the same time interval as the treatment period. But as for ethical considerations, the comparison is limited to the only one time point when the treatment ended. No significant differences in the airway size were detected, indicating that the effect of orthodontic extraction treatment is of little clinical significance.

The present study found an unchanged 3D upper airway with decreased sagittal dimensions in adults with Class II and hyperdivergent skeletal pattern, and investigated the possible reasons as self-regulation of the airway morphology. The effect of the morphological change on the respiratory function remains unknown, and whether such effect is stable is another important question. Strictly control of the inclusive and exclusive criteria greatly limits the sample size in the present study. The small sample size leads to a decreased power of test, and thus an increased probability of type II error. These limitations, as well as the non-randomized design, prevent the generalization of the findings. Therefore, further prospective investigations with a larger sample size, greater length of follow-up, and assessment of the effect on respiratory function will allow a better understanding.

## Conclusions

The null hypothesis is not rejected.

1. The changes of the airway volume, height, and cross-sectional area after orthodontic extraction treatment were not significant in adult patients with Class II and hyperdivergent pattern.
2. The changes of the sagittal airway dimensions after orthodontic extraction treatment were significant in the middle and inferior parts of the upper airway.
3. The morphology of the airway cross sections was compressed at the anteroposterior dimension with unchanged area after orthodontic extraction treatment in the middle and inferior part of the upper airway.
4. There was no significant difference in the airway size between the post-treatment patients and untreated matched controls.

## Supporting Information

S1 File Raw data of the craniofacial measurements of the treated patients. (XLS)

S2 File Raw data of the airway measurements of the treated patients. (XLS)

S3 File Raw data of the airway measurements of the untreated matched controls. (XLSX)
